# iPiDA-SWGCN: Identification of piRNA-disease associations based on Supplementarily Weighted Graph Convolutional Network

**DOI:** 10.1371/journal.pcbi.1011242

**Published:** 2023-06-20

**Authors:** Jialu Hou, Hang Wei, Bin Liu

**Affiliations:** 1 School of Computer Science and Technology, Beijing Institute of Technology, Beijing, China; 2 School of Computer Science and Technology, Xidian University, Xi’an, Shaanxi, China; 3 Advanced Research Institute of Multidisciplinary Science, Beijing Institute of Technology, Beijing, China; ., CANADA

## Abstract

Accurately identifying potential piRNA-disease associations is of great importance in uncovering the pathogenesis of diseases. Recently, several machine-learning-based methods have been proposed for piRNA-disease association detection. However, they are suffering from the high sparsity of piRNA-disease association network and the Boolean representation of piRNA-disease associations ignoring the confidence coefficients. In this study, we propose a supplementarily weighted strategy to solve these disadvantages. Combined with Graph Convolutional Networks (GCNs), a novel predictor called iPiDA-SWGCN is proposed for piRNA-disease association prediction. There are three main contributions of iPiDA-SWGCN: (i) Potential piRNA-disease associations are preliminarily supplemented in the sparse piRNA-disease network by integrating various basic predictors to enrich network structure information. (ii) The original Boolean piRNA-disease associations are assigned with different relevance confidence to learn node representations from neighbour nodes in varying degrees. (iii) The experimental results show that iPiDA-SWGCN achieves the best performance compared with the other state-of-the-art methods, and can predict new piRNA-disease associations.

## Author summary

PIWI-interacting RNAs (piRNAs) are a kind of small non-coding RNAs with approximately 23–36 nucleotides in length, showing critical impact on various biological processes. It is crucial to develop computational methods for identifying piRNA-disease associations. Although several computational methods have contributed to the piRNA-disease association detection, there are still two main limitations for the further improvement of piRNA-disease association prediction: (i) The high sparsity of piRNA-disease associations prevent the predictors to accurately infer potential associations. (ii) Most existing methods focus on the Boolean connectivity of piRNA-disease associations, ignoring the correlation information with different confidence. To solve aforementioned limitations, we propose a supplementarily weighted strategy in this study. Combined with Graph Convolutional Networks (GCNs), a novel predictor named iPiDA-SWGCN is proposed for piRNA-disease association prediction. The supplementarily weighted strategy for piRNA-disease association complement cannot only enrich network structure information, but also provide the association confidence information for GCN to learn node representations. As a result, the evaluation results show that iPiDA-SWGCN can effectively detect missing piRNA-disease associations, and outperforms the other state-of-the-art methods.

## Introduction

PIWI-interacting RNAs (piRNAs) belong to a category of small non-coding RNAs composed of approximately 23–36 nucleotides [1,2], showing a critical impact on various biological processes by regulating gene expression at epigenetic and post-transcriptional levels [3,4]. For example, piRNAs bind to piwi proteins for regulating transposon silencing, genome rearrangement, spermiogenesis, germ stem-cell maintenance, etc [5,6].

Emerging evidence indicate that piRNAs participate in many disease genesis and prognosis [7–9]. For example, piR-36712 is downregulated in breast cancer by suppressing cell proliferation, invasion and migration through the combination with SEPW1P RNA [10]. The piR-651 shows upregulated expression in gastric cancer, and tends to be associated with TNM stages [11]. Several studies highlight that piRNAs can be viewed as potential biomarkers of disease diagnosis and prognosis for effective therapeutic project design [12,13]. Therefore, developing computational methods has great significance for identifying piRNA-disease associations.

Several computational methods have been proposed to predict the associations between non-coding RNA s and diseases. These methods usually rely on network link [14], recommendation system [15], matrix completion [16], classical machine learning [17] and deep learning [18]. However, research on the detection of piRNA-disease associations is still in its preliminary stages. To unravel the complex interactions between piRNAs and diseases, several computational methods for predicting piRNA-disease associations have been proposed [19–23]. For example, iPiDi-PUL [19] and iPiDA-sHN [20] were proposed to predict piRNA-disease association based on positive-unlabeled learning. APDA [21] and iPiDA-GBNN [22] employed a stacked auto-encoder to extract piRNA-disease pair features, and then it was trained with random forests and Gradient Boosting Neural Network respectively to predict new piRNA-disease associations. DFL-PiDA [23] combined convolutional de-noising auto-encoder and extreme learning machine to identify potential associations. iPiDA-LTR [24] employed a ranking framework to integrate several component methods for piRNA-disease association detection. To further improve the representation ability of association, iPiDA-GCN [25] designed Asso-GCN and Sim-GCN models for iteratively extracting features for piRNAs and diseases.

Although the aforementioned methods have contributed to the piRNA-disease association detection, there are two main limitations for the further improvement of piRNA-disease association prediction: (i) The high sparsity of piRNA-disease associations. For example, there are only about 5% piRNA-disease associations with experimental validation in piRDisease v1.0 [26] and MNDR v3.0 [27]. The lack of association information will prevent the predictors to accurately infer the piRNA-disease associations. (ii) The Boolean associations between piRNAs and diseases. Most of the existing methods for piRNA-disease association detection utilize Boolean values to denote whether a piRNA is related with a disease or not during the training process. However, piRNAs are related with diseases with different probabilities. Boolean associations only focusing on the connectivity will ignore the confidence information.

**Fig 1:**
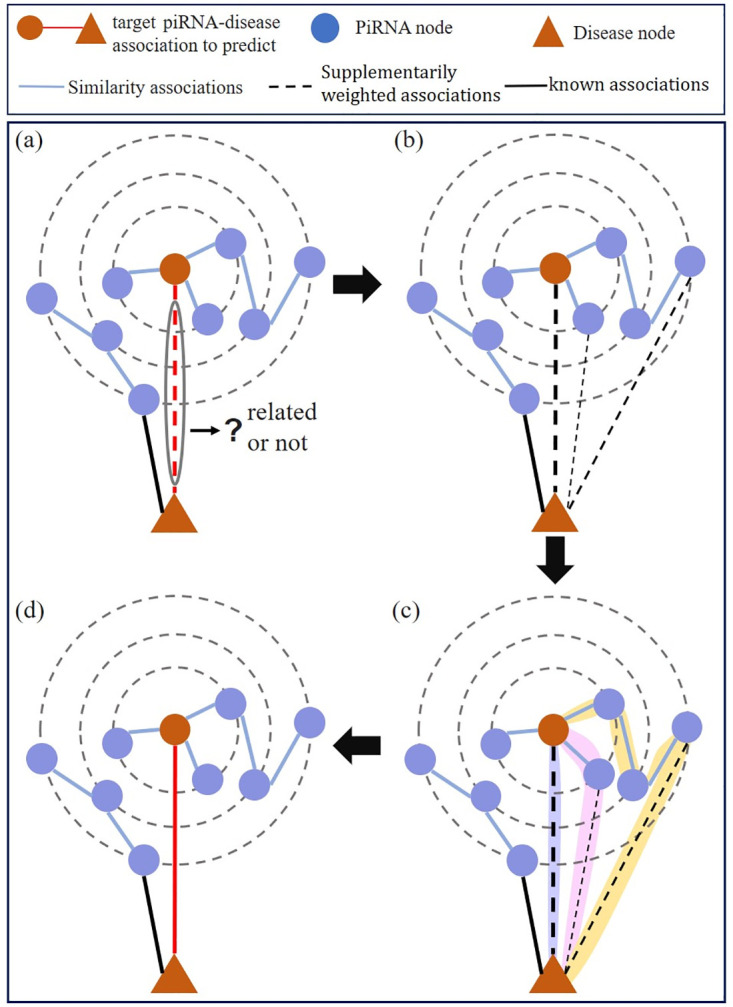
The effectiveness of the supplementarily weighted strategy. (a) The original piRNA-disease network is constructed based on similarity information and piRNA-disease associations. (b) PiRNA-disease network with supplementarily weighted associations. (c) Three feasible paths for piRNA-disease association detection, highlighted in purple, pink and yellow shadows. (d) The final prediction results for the target association. Integrating the inferences from three feasible paths, the final prediction result is obtained and denoted as the red line.

To solve the above limitations, we propose a supplementarily weighted strategy to enrich the topology structure information of piRNA-disease network so as to provide more information for piRNA-disease association detection. As shown in Fig 1, our goal is to infer whether the target piRNA is associated with the target disease or not. In the original network, as the lack of link information, it is difficult to detect whether the target association exists or not (Fig 1a). Then three weighted piRNA-disease associations are preliminarily supplemented (Fig 1b). Therefore, three feasible paths can be generated to infer the association probability (Fig 1c). Finally, the prediction results are obtained by integrating different inference results based on their feasible paths (Fig 1d). As a result, supplementarily weighted associations can enrich the connectivity information, and yield more possible paths to comprehensively predict the relationship between piRNAs and diseases.

With the development of representation learning, Graph Convolutional Network (GCN) [28] is proposed to extend the CNN for graph-structured data, and achieves powerful ability of representation learning by capturing rich structural information [29]. In this paper, we combine the supplementarily weighted strategy and GCN, and propose a novel predictor named iPiDA-SWGCN for piRNA-disease association detection with three major contributions: (i) The supplementarily weighted matrix with high-quality computed by machine learning methods can provide more information for the original sparse piRNA-disease association network, based on which GCN can aggregate more neighbour node information for expressive feature learning. (ii) The Boolean associations are replaced by weighted associations with the computed relevance confidence. With the piRNA-disease associations assigned with different weights, GCN can learn node representations from neighbour nodes in varying degrees. (iii) The evaluation results indicate that iPiDA-SWGCN has the ability to effectively detect missing piRNA-disease associations, and shows superior performance than the other state-of-the-art methods.

## Materials and methods

### Datasets

A recently constructed database MNDR v3.0 (http://www.rnadisease.org/) [27] records different categories of ncRNA-disease associations. After removing duplicated associations and extracting human-related piRNA-disease associations, 11981 experimentally validated piRNA–disease associations are selected to construct the dataset with 10149 piRNAs and 19 diseases.

In this study, machine learning predictors are trained with two phases: (i) Training several basic classifiers to compute the weights of unknown piRNA-disease associations. (ii) Training GCN to capture piRNA and disease features. In the first phase, the dataset D_*all*_ is split into a benchmark dataset $D_{ben}^{1}$ and an independent dataset $D_{ind}^{1}$. We train several basic predictors on the benchmark dataset, and then utilize the trained predictors to score the associations in the independent dataset. The datasets can be formulated as:

$$\left\{\begin{array}{c} {\text{D}}_{all}={\text{D}}_{ben}^{1}+{\text{D}}_{ind\;}^{1}\\ {\text{D}}_{all}={\text{D}}_{all}^{+}+{\text{D}}_{all\;}^{-}\\ {\text{D}}_{ben}^{1}={\text{D}}_{ben}^{1+}+{\text{D}}_{ben\;}^{1-}\\ {\text{D}}_{ind}^{1}={\text{D}}_{ind}^{1+}+{\text{D}}_{all\;}^{-} \end{array}\right.$$ (1)

where $D_{all}^{+}$ is the positive set with 11981 known piRNA-disease associations. $D_{all}^{-}$ contains 180850 unknown piRNA-disease associations. $D_{ben}^{1+}$ and $D_{ben}^{1-}$ are randomly selected from D_*all*_ with the equivalent number of piRNA-disease associations, representing the positive and negative subset of $D_{ben}^{1}$, respectively. In order to assign weights for all unknown piRNA-disease associations, the negative independent set $D_{all}^{-}$ contains all unknown associations. The positive independent set $D_{ind}^{1+}$ are constructed by randomly selecting 20% positive associations in $D_{all}^{+}$.

In the second training phase, we divide the dataset following the previous studies [30,31]:

$$\left\{\begin{array}{c} {\text{D}}_{all}={\text{D}}_{ben}^{2}+{\text{D}}_{ind\;}^{2}\\ {\text{D}}_{ben}^{2}={\text{D}}_{ben}^{2+}+{\text{D}}_{ben\;}^{2-}\\ {\text{D}}_{ind}^{2}={\text{D}}_{ind}^{2+}+{\text{D}}_{ind\;}^{2-} \end{array}\right.$$ (2)

where the positive benchmark dataset $D_{ben}^{2+}$ contains 80% of positive piRNA-disease associations randomly selected from $D_{all}^{+}$, and the remaining positive associations constitute the positive independent dataset $D_{ind}^{2+}$. The negative independent dataset $D_{ind}^{2-}$ are randomly selected from $D_{all}^{-}$ with the equal size of $D_{ind}^{2+}$, and the rest of negative samples constitute the sub-dataset $D_{ben}^{2-}$.

To prevent overestimating the performance of the proposed method, the piRNA-disease associations in the independent dataset for model evaluation are removed from the training phases:

$$\left\{\begin{array}{c} {\text{D}}_{ben}^{1+}\cap {\text{D}}_{ind}^{1+}=\emptyset \\ {\text{D}}_{ben}^{2}\cap {\text{D}}_{ind}^{2}=\emptyset \\ {\text{D}}_{ben}^{1}\cap {\text{D}}_{ind}^{2}=\emptyset \end{array}\right.$$ (3)

### Method overview

**Fig 2:**
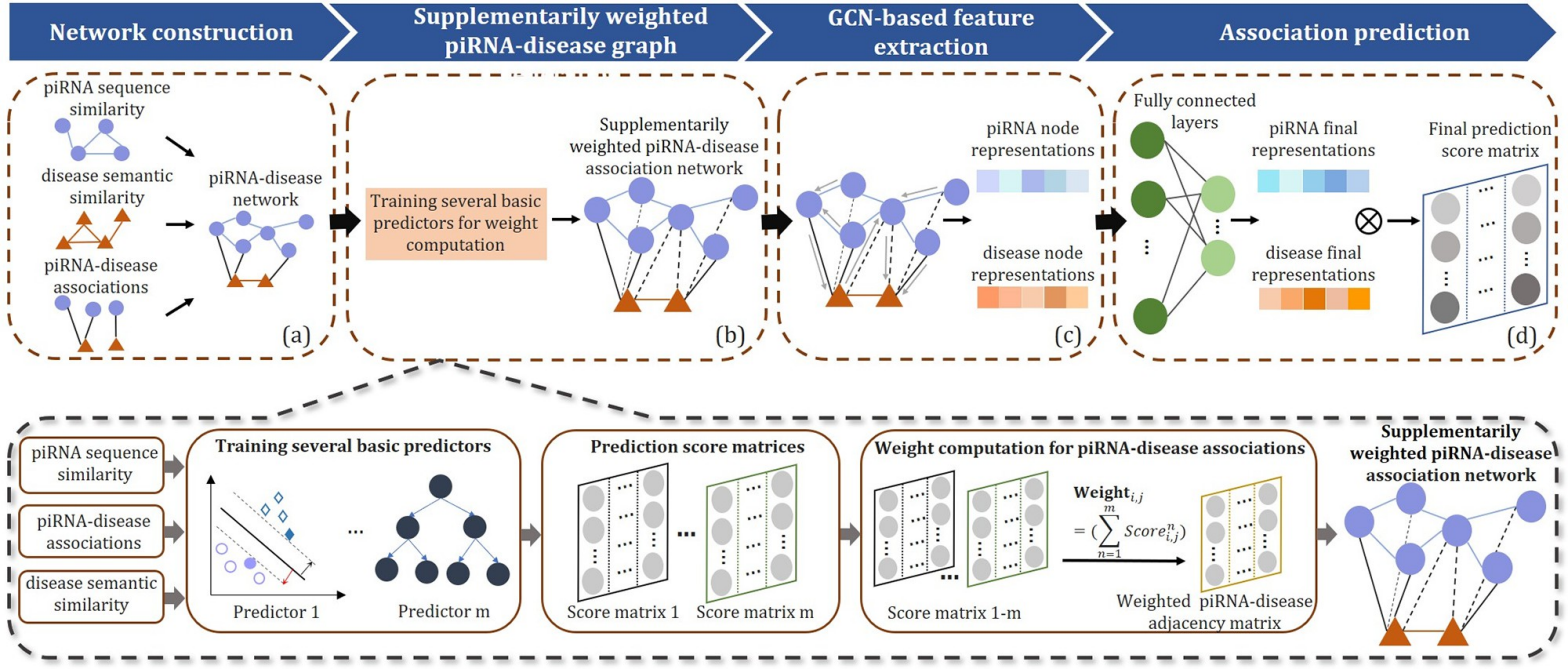
The flowchart of iPiDA-SWGCN. (a) piRNA-disease network is constructed by integrating piRNA sequence similarity, disease semantic similarity and piRNA-disease associations. (b) The supplementarily weighted piRNA-disease network is generated based on the proposed supplementarily weighted strategy and the weights are computed via several basic predictors. (c) piRNA and disease node features are extracted by performing GCN on the supplementarily weighted piRNA-disease network. (d) Fully connected layers and inner production are employed to predict final association scores.

In this study, we propose a novel method named iPiDA-SWGCN for piRNA-disease association detection. The overall process of iPiDA-SWGCN is shown in Fig 2 with four parts: (i) Network construction (Fig 2a). A heterogeneous piRNA-disease network is constructed by integrating piRNA and disease information; (ii) Supplementarily weighted piRNA-disease network generation (Fig 2b). Different supplementary weights are assigned to unknown piRNA-disease pairs based on the scores computed by several predictors; (iii) GCN-based feature extraction (Fig 2c). In this section, GCN is performed on the supplementarily weighted piRNA-disease network to capture the structural information, and extract feature representations of piRNAs and diseases. (iv) Association prediction (Fig 2d). Finally, we use the fully connected layers for dimension reduction and inner product operation so as to calculate the association scores between piRNAs and diseases.

### Network construction

In this section, we construct a heterogeneous piRNA-disease network denoted as G = {E_*piR*−*disease*_, E_*piR*−*piR*_, E_*disease*−*disease*_, V_*piR*_, V_*disease*_}. In detail, three types of edges and two types of nodes are included in the piRNA-disease network. The three kinds of edges are piRNA-disease associations, piRNA-piRNA edges and disease-disease edges, represented as E_*piR*−*disease*_, E_*piR*−*piR*_ and E_*disease*−*disease*_, respectively. E_*piR*−*disease*_ are the original piRNA-disease associations derived from the database MNDR v3.0. The other two kinds of edges are obtained based on the similarity between homogeneous biological entities. V_*piR*_ and V_*disease*_ represent the nodes of piRNAs and diseases. The specific calculation of above edge and node representations will be introduced in the following sections.

#### PiRNA-disease associations

The adjacency matrix **A**_PD_ represents the associations for each pair of piRNA-disease, denoted as:

$${\text{A}}_{\text{PD}}=\left[\begin{array}{ccc} \begin{array}{cc} a_{1,1} & a_{1,2}\\ a_{2,1} & a_{2,2} \end{array} & \cdots & \begin{array}{c} a_{1,n}\\ a_{2,n} \end{array}\\ \vdots \;\vdots & \ddots & \vdots \\ \begin{array}{cc} a_{m,1} & a_{m,2} \end{array} & \cdots & a_{m,n} \end{array}\right]$$ (4)

where *m* and *n* are the number of piRNAs and diseases, respectively. The element *a*_*i*,*j*_ is 1, if the association between *i-*th piRNA and the *j-*th disease is confirmed with experimental verification, otherwise *a*_*i*,*j*_ = 0.

#### PiRNA-piRNA sequence similarity

For each pair of piRNAs, the sequence similarity is calculated, where the sequence information is downloaded from piRBase v3.0 (http://bigdata.ibp.ac.cn/piRBase/) [32]. PiRNA-piRNA sequence similarity are calculated via Smith-Waterman local alignment algorithm [33], which is highly sensitive and can detect subtle similarities between sequences with low identity in a robust and accurate manner [34,35]. We compute the sequence similarity score for given pair of piRNAs as [33]:

$${\text{P}}_{\text{seq}}\left(p_{i},p_{j}\right)=\frac{\text{SW}\left(p_{i},p_{j}\right)}{\sqrt{\text{SW}\left(p_{i},p_{i}\right)\times \text{SW}\left(p_{j},p_{j}\right)}}$$ (5)

where SW(*p*_*i*_, *p*_*j*_) denotes the sequence alignment score between *p*_*i*_ and *p*_*i*_ calculated by Smith-Waterman alignment algorithm.

#### Disease-disease semantic similarity

Disease semantic similarity has been extensively used in ncRNA-disease association detection [36–39]. Directed Acyclic Graph (DAG) describes the relationship among different disease terms. Disease semantic similarity can be calculated based on the Disease Ontology (DO) descriptors in DAG [40–42]. The Directed Acyclic Graph (DAG) based algorithm not only uses a consistent standard to make the calculated disease similarity uniform and comparable but also is capable of fully capturing complex relationships between diseases and better represent the disease space and the interconnection of diseases. In this study, we adopt one of the most effective semantic similarity measurements [41,43,44] to compute the disease semantic similarity. For disease *d*_*i*_ and disease *d*_*j*_, the disease semantic similarity **D**_sem_(*d*_*i*_, *d*_*j*_) is calculated by [44]:

$${\text{D}}_{\text{sem}}\left(d_{i},d_{j}\right)=\frac{\sum_{t\in {\text{T}}_{i}⋂{\text{T}}_{j}}\left({\text{S}}_{d_{i}}\left(t\right)+{\text{S}}_{d_{j}}\left(t\right)\right)}{\sum_{t\in {\text{T}}_{i}}{\text{S}}_{d_{i}}\left(t\right)+\sum_{t\in {\text{T}}_{j}}{\text{S}}_{d_{j}}\left(t\right)}$$ (6)

$$\left\{\begin{array}{ll} {\text{S}}_{d_{i}}\left(t\right)=\text{max}\left\{\theta *{\text{S}}_{d_{i}}\left(t\prime \right)|t^{\prime }\;\in \;\text{children}\;\text{of}\;\left(t\right)\right\} & \text{if}\;d_{i}\;\neq \;t\\ {\text{S}}_{d_{i}}\left(t\right)=1 & \text{otherwise} \end{array}\right.$$ (7)

where T_*i*_ is composed of all subterms in the DAG of disease *d*_*i*_, ${\text{S}}_{d_{i}}\left(t\right)$ indicates the semantic contribution of disease *t* ∈ T_*i*_ to the *i-*th disease according to [44]. *θ* is the semantic contribution factor which is set as 0.5 following [44].

#### Node feature extraction

The row vector of similarity matrix **P**_seq_ or **D**_sem_ can serve as the feature vector for a piRNA or disease. However, they ignore the connectivity information, especially for non-neighboring and higher-order connected nodes. Therefore, in order to introduce the global topology information of similarity networks, we further apply random walk with restart (RWR) [45] on **P**_seq_ and **D**_sem_ to extract piRNA and disease features. The feature generation for node *i* based on RWR can be formulated as [45]:

$${\text{F}}_{i}^{k+1}=\left(1-\alpha \right){\text{F}}_{i}^{k}\text{S}+\alpha y_{i}$$ (8)

where ${\text{F}}_{i}^{k}$ is the row vector of node *i*, whose elements indicate the probabilities of walking from node *i* to all the other homogeneous nodes after *k* steps. **S** is the probability transition matrix obtained from similarity matrix (**P**_seq_ or **D**_sem_) with row-wise normalization, *y*_*i*_ is a one-hot vector representing the initial probability vector of node *i*, and *α* is the restart probabilities. Finally, we obtain ${\text{F}}_{i}^{\infty }$ as the feature descriptor of node *i*.

### Supplementarily Weighted Graph Convolutional Network (SWGCN)

Many unknown pairs lead to the high sparsity of piRNA-disease association network. GCN performing on such a network may lead to performance degradation because of limited neighbor information aggregation. To overcome this problem, we propose the Supplementarily Weighted Graph Convolutional Network (SWGCN). Firstly, the supplementarily weighted adjacency matrix is computed to achieve an informative piRNA-disease association network with high quality, and then GCN is adopted to capture hidden structure information for feature learning.

#### Weights computation for piRNA-disease association

In this section, we define the supplementarily weighted adjacency matrix in SWGCN for piRNA-disease association completion as follows:

*Definition 1*. *Supplementarily weighted adjacency matrix*. Given a network G = {V, E}, we train several basic machine-learning-based predictors to compute the average relevance scores for unknown associations among nodes. Specifically, *w*_*i*,*j*_ represents the average score of edge *e*_*i*,*j*_ between node *n*_*i*_ and node *n*_*j*_, and the adjacency matrix of the completed network can be denoted as ${\text{A}}_{\text{PD}}^{\prime }\in {\text{R}}^{m\times n}$, where ${\text{A}}_{\text{PD}}^{\prime }\left(i,j\right)=\left\{w_{i,j},1\right\}$, i.e., if **A**_PD_(*i*, *j*) = 0, then ${\text{A}}_{\text{PD}}^{\prime }\left(i,j\right)=w_{i,j}$, otherwise ${\text{A}}_{\text{PD}}^{\prime }\left(i,j\right)=1$.

To improve the quality of SWGCN, 15 different basic predictors based on Random Forest [46], Support Vector Machine (SVM) [47], Gradient Boosting Decision Tree (GBDT) [48] are trained for weight computation. It should be noted that we train various predictors on different training sets, where the positive training set $D_{ben}^{1+}$ keeps the same while the negative training sets $D_{ben}^{1-}$ are randomly selected from $D_{all}^{-}$ for five times. For a pair of piRNA *p*_*i*_ and disease *d*_*j*_, the element ${\text{A}}_{\text{PD}}^{\prime }\left(i,j\right)$ is formulated as:

$${\text{A}}_{\text{PD}}^{\prime }\left(i,j\right)=\left\{\begin{array}{c} 1\;\text{if}\;\left(p_{i},d_{j}\right)\in {\text{D}}_{ben}^{1+}\\ \frac{\sum_{k=1}^{5}(s_{\text{SVM}}^{k}+s_{\text{RF}}^{k}+s_{\text{GBDT}}^{k})}{15}\;\text{otherwise} \end{array}\right.$$ (9)

where $s_{SVM}^{k}$ denotes the prediction score computed by the *k-*th SVM classifier. 15 predictors are employed to score the unknown associations, and the average scores are set as the weights for unknown associations. Finally, the weighted edges are added into the original heterogeneous network to generate the supplementarily weighted piRNA-disease network.

#### Feature learning based on Graph Convolutional Network

GCN has been widely used for capturing graph structural information, and aggregating neighbour information so as to extract the node features [49,50]. After assigning weights to piRNA-disease associations, GCN is performed on the supplementarily weighted piRNA-disease network to learn the feature representations of different nodes by information aggregation from neighbours. The node embedding **H**^*l*^ learned by GCN in the *l*-th layer can be formulated as [28]:

$${\text{H}}^{l}=\sigma \left({\tilde{\text{D}}}^{-\frac{1}{2}}\tilde{{\text{A}}_{all}}{\tilde{\text{D}}}^{-\frac{1}{2}}{\text{H}}^{l-1}{\text{W}}^{l-1}\right)$$ (10)

where

$${\text{A}}_{all}=\left[\begin{array}{cc} {\text{P}}_{\text{seq}} & {\text{A}}_{\text{PD}}^{\prime }\\ {\text{A}}_{\text{PD}}^{\prime }{}^{\text{T}} & {\text{D}}_{\text{sem}} \end{array}\right]$$ (11)

$$\tilde{{\text{A}}_{all}}=\text{I}+{\text{A}}_{all}$$ (12)

$$\tilde{\text{D}}\left(i,i\right)=\sum_{j}\tilde{{\text{A}}_{all}}\left(i,j\right)$$ (13)

where the adjacency matrix **A**_***all***_ ∈ R^(m+n)×(m+n)^ indicates the association adjacency matrix for the whole network, and $\tilde{A_{all}}$ is **A**_***all***_ added with self-loop. $\tilde{D}$ denotes the degree matrix of $\tilde{A_{all}}$, **W** represents the trainable parameters of GCN model, σ(·) denotes the nonlinear activation function. **H**^*l*^ ∈ R^(m+n)×c^ is initialized by concatenating the piRNA and disease representations, where c denotes the common dimension of piRNA and disease features obtained by the dense layer. It is denoted that the batch norm layer is added before each dense layer and convolution layer to alleviate the problem of vanishing or exploding gradients and speed up the convergence. The batch norm layer can standardize the mean and variance of the input to each layer based on the statistics computed over a batch of training examples to make the distribution of each layer’s input relatively stable, and further reduce the risk of overfitting.

**Fig 3:**
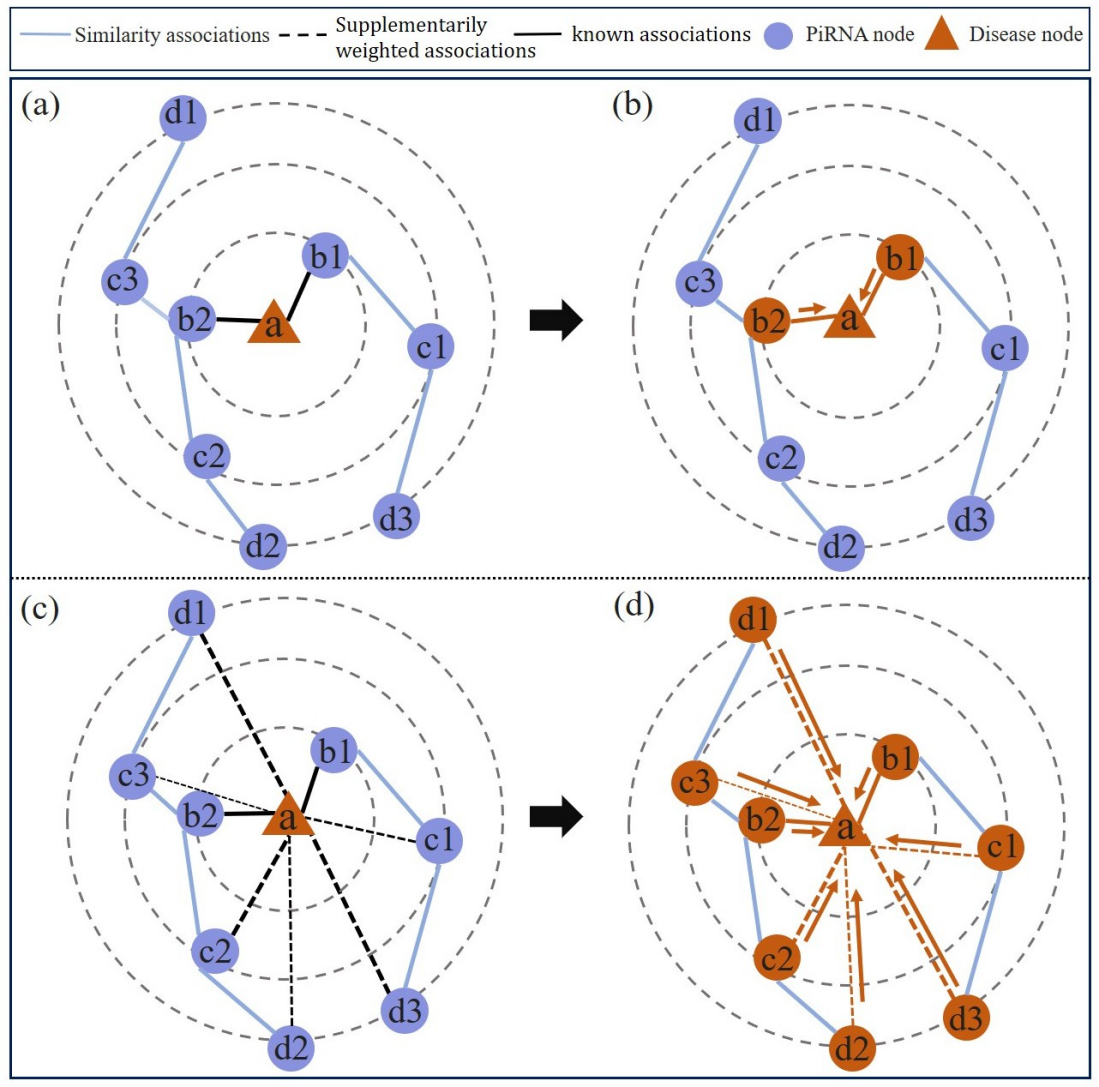
Comparison of performing GCN on different networks to aggregate neighbor information. **Fig 3a and 3b** show the receptive field of 1-layer of GCN on the original piRNA-disease association network, while **Fig 3c and 3d** show the receptive field of 1-layer GCN on the supplementarily weighted association network.

Compared with the original piRNA-disease network, GCN can effectively convolve more useful neighbor information with supplementarily weighted edges. Fig 3 shows the comparison of performing GCN on different networks. Take the disease node *a* as an example, on the original piRNA-disease network, the first layer of GCN updates the feature representation of node *a* by aggregating the features of its first-order neighbor node *b*1 and *b*2 (Fig 3a and 3b), failing to capture the indirectly connected node information of *c*1, *c*2, *c*3, *d*1, *d*2, *d*3. In contrast, the node representation of *a* can be learned by aggregating all piRNA node information with different concerns on the supplementarily weighted piRNA-disease association network (Fig 3c and 3d). As a result, SWGCN is able to capture deeper and wider neighbor information for informative feature learning.

### Association prediction

After obtaining the feature representation learned by GCN, the full connection networks with three layers are constructed to separately reduce the dimensions of representations for piRNAs and diseases. The probability of piRNA *p*_*i*_ associated with disease *d*_*i*_ is calculated by inner production as:

$${\text{U}}_{i,j}={\text{f}}_{p_{i}}^{\text{'}}{\text{f}}_{d_{j}}^{\text{'}}{}^{T}$$ (14)

where $f_{p_{i}}^{'}$ and $f_{d_{j}}^{'}$ denote the feature representation of piRNA *p*_*i*_ and disease *d*_*i*_ after dimensionality reduction, and **U** is the predicted final score matrix for the associations between piRNAs and diseases. It should be noted that we conduct Batch Normalization (BN) [51] following each convolution layer to mitigate internal covariate shift and increase the stability.

We utilize the mean square error as the loss function which minimizes the Frobenius norm of the difference between prediction score matrix **U** and the label matrix **A**_PD_. Nevertheless, the high sparsity of piRNA-disease association matrix may cause prediction bias to the unknown associations. To alleviate this problem, we adopt *β*-enhanced loss function [52] that enlarges the margin between the real label matrix **A**_PD_ and predicted score matrix **U** with hyper-parameter *α*. The loss function is formulated as:

$$\text{Loss}=||{\hat{\text{A}}}_{\text{PD}}-\text{U}|{|}_{F}^{2}+\mu ||\text{W}|{|}_{2}^{2}$$ (15)

where

$${\hat{\text{A}}}_{PD}=\left\{\begin{array}{c} 0\;\text{if}\;{\text{A}}_{\text{PD}}\left(i,j\right)=0\;\text{or}\;{\text{A}}_{\text{PD}}\left(i,j\right)\in {\text{D}}_{ind}^{2}\\ \beta \;\text{otherwise} \end{array}\right.$$ (16)

where ${\hat{A}}_{PD}$ indicates the enhanced association matrix. **W** denotes the trainable parameters. *μ* is a decay factor controlling the regularization term of **W** to prevent overfitting.

### Performance evaluation

Two metrics are employed to comprehensively evaluate the predictor performance, including AUPR (area under the precision recall curve) [53] and AUC (area under the receiver operating characteristics curve) [54]. AUC measures the sensitivity and specificity of the model [55], and AUPR can avoid the impact of imbalanced data sets, and comprehensively reflect the quality of predictions [56].

## Results and discussion

### Combination of basic methods can improve the quality of SWGCN

**Fig 4:**
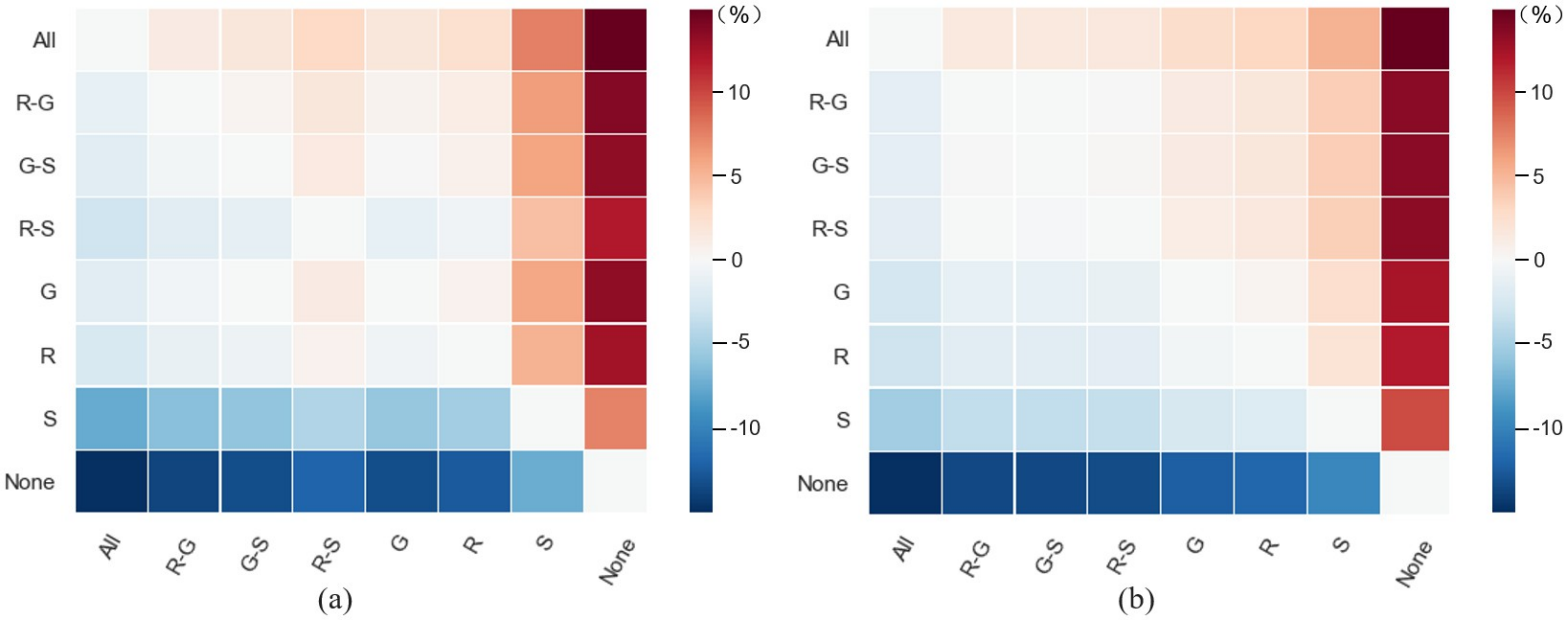
Performance comparison of different basic method combinations. (a) and (b) show the difference values in terms of AUC and AUPR among different predictors, respectively. The difference values are computed by the scores obtained from the method labelled at *y*-axis minus the scores obtained from the method labelled at *x*-axis.

In this study, three basic machine learning methods (RF, SVM and GBDT) are used to compute weights for the unknown piRNA-disease pairs. To analyze their contribution to weighting associations in the proposed model, we compare the predictors based on different basic methods and their combinations. Table 1 lists the results, and their performance differences are shown in Fig 4, from which we can draw the following conclusions: (i) Assigning weights to associations indeed contributes to performance improvement. (ii) It is not surprising that integrating several complementary methods for weights computation can effectively improve the performance compared with using a single basic method. (iii) iPiDA-SWGCN outperforms all the other methods because it integrates all the basic methods to compute weights so as to assign weights to piRNA-disease associations with better performance.

**Table 1:** Performance comparison between iPiDA-SWGCN and predictors based on different basic methods and their combinations for weighing associations on $D_{ind}^{2}$.

Methods^a^	AUC	AUPR
iPiDA-SWGCN (All)	**0.8178**	**0.8151**
R-G	0.8039	0.8026
G-S	0.8043	0.7985
R-S	0.8028	0.7851
G	0.7925	0.7978
R	0.7883	0.7914
S	0.7688	0.7397
None	0.6755	0.6654

^a^ The basic methods RF, SVM and GBDT are denoted as ‘R’, ‘S’ and ‘G’ respectively. ‘R-S’ indicates that we employ RF and SVM to weight the associations, and then feed the weighted network into GCN, ‘G-S’ indicates that GBDT and SVM are used to compute weights, and then GCN is employed. Other predictors are notated with the same rule. ‘All’ denotes that all the basic methods are adopted for weight computation, and ‘None’ denote no basic method is used, GCN is performed on the original piRNA-disease network.

### Impact of different association adjacency matrices on the performance of iPiDA-SWGCN

Previous studies represent the piRNA-disease associations as Boolean values, where the element in the adjacency matrix is 1 if the association in the network is known, otherwise it is 0. In this study, we compute weights for unknown associations in the network instead of 0. To illuminate the effectiveness and necessity of assigning weights instead of Boolean values for unknown edges, we perform experiments on five networks with different edge types. The experimental results are listed in Table 2, from which we can see that the model with the weighted network is superior to those with the network with added Boolean piRNA-disease edges. The reason is that assigning weights to associations cannot only enrich more network structural information, but also can provide association confidence information for GCN so as to learn the expressive node representation. In contrast, Boolean edges only indicate whether the associations exist or not.

**Table 2:** The performance comparison of iPiDA-SWGCN based on different adjacency matrices on $D_{ind}^{2}$.

Methods	AUC	AUPR
iPiDA-SWGCN_Bool10^a^	0.7361	0.7629
iPiDA-SWGCN_Bool20^b^	0.7357	0.7691
iPiDA-SWGCN_Bool30^c^	0.7313	0.7569
iPiDA-SWGCN_Bool40^d^	0.7219	0.7346
iPiDA-SWGCN	**0.8178**	**0.8151**

^a,b,c,d^ The unknown piRNA-disease pairs are ranked according to their prediction scores computed by basic predictors. The methods with suffix ‘_Bool10’, ‘_Bool20’, ‘_Bool30’ and ‘_Bool40’ denote that GCN is performed on each supplementarily weighted matrix with bool weights assigned, where the top 10%, 20%, 30% and 40% unknown associations with the highest predicted scores are computed by several base predictors, then these associations are viewed as positive samples and supplemented into the original piRNA-disease networks.

### Performance comparison among different methods

To illuminate the effectiveness of iPiDA-SWGCN for identifying piRNA-disease associations, we conducted a performance comparison of our method with five state-of-the-art approaches, including iPiDi-PUL [19], iPiDA-sHN [20], iPiDA-LTR [24], iPiDA-GCN [25] and piRDA [57]. The web servers or source codes of all these methods are accessible, enabling unbiased evaluation of their performance. The experimental results are displayed in Table 3, from which we can concluded that iPiDA-SWGCN outperforms all the other methods. iPiDA-SWGCN is superior to iPiDA-GCN by 10.29% and 11.15% in terms of AUC and AUPR, respectively. The performance improvement can be attributed to the fact that iPiDA-SWGCN is able to capture more expressive node representations by aggregating more neighbor information from the supplementarily weighted network.

**Table 3:** Performance comparison of different methods on $D_{ind}^{2}$.

Methods^a^	AUC	AUPR
iPiDi-PUL^b^	0.6653	0.6550
iPiDA-sHN^c^	0.5226	0.5203
iPiDA-LTR^d^	0.6147	0.6641
iPiDA-GCN^e^	0.7149	0.7036
piRDA^f^	0.4939	0.5116
**iPiDA-SWGCN** ^g^	**0.8178**	**0.8151**

^a^All these predictors are trained with $D_{ben}^{2}$. The results of iPiDi-PUL [19], iPiDA-sHN [20] and iPiDA-GCN are obtained from [25]. The result of iPiDA-LTR [24] is obtained by in-house implementation;

^b^Results obtained with parameters n_components = 200, n_estimators = 150, max_features = 0.2 and number of ensemble learner = 5;

^c^Results obtained with parameters C = 1.0, kernel = ‘rbf’ and gamma = 1;

^d^Results obtained with parameters tree = 120, shrinkage = 0.22 and leaves = 3;

^e^Results obtained with parameters epoch = 2000, learning rate = 0.001, weight decay factor = 1.0 and learn rate decay = 0.2;

^f^The results were generated using the web server of piRDA (http://nsclbio.jbnu.ac.kr/tools/piRDA/). Since piRDA was developed based on the piRDisease dataset, it is limited to predicti associations related to only 13 diseases in $D_{ind}^{2}$. Therefore, we only evaluated the prediction results for these associations;

^g^Results obtained with parameters epoch = 1000, learning rate = 0.001, weight decay factor = 1.0 and learn rate decay = 0.2.

### Visualization of predicted associations by iPiDA-SWGCN

In order to visually illustrate the effectiveness of the supplementarily weighted network used in iPiDA-SWGCN, the prediction results of GCN performing on different networks are compared. We take two piRNA-disease associations for further analysis, including <piR-hsa-22710, Parkinson’s disease> and <piR-hsa-28405, renal cell carcinoma>. Parkinson’s Disease (PD) is a widely prevalent neurodegenerative disorder. The gross pathological findings reveal significant damage to dopaminergic neurons in the midbrain’s substantia nigra (SN), leading to dopamine deficiency in the nerve terminals located in the basal ganglia [58]. PiR-has-22710 has length of 30nt and is downregulated in PD-patient tissue samples [59]. Renal cell carcinoma (RCC) is a prevalent cancer, ranking sixth in incidence in men and tenth in women globally [59]. Recent research suggests that the expression levels of piRNA are linked to the histological grade, pathological features of RCC, and patient survival. Multiple studies have demonstrated that piRNAs show differentially expressed in benign versus malignant renal tumor tissues [59,60]. PiR-has-28405 is a type of Homo sapiens piRNA with length of 32nt, showing about 4-fold downregulation in renal tumor tissue [60].

**Fig 5:**
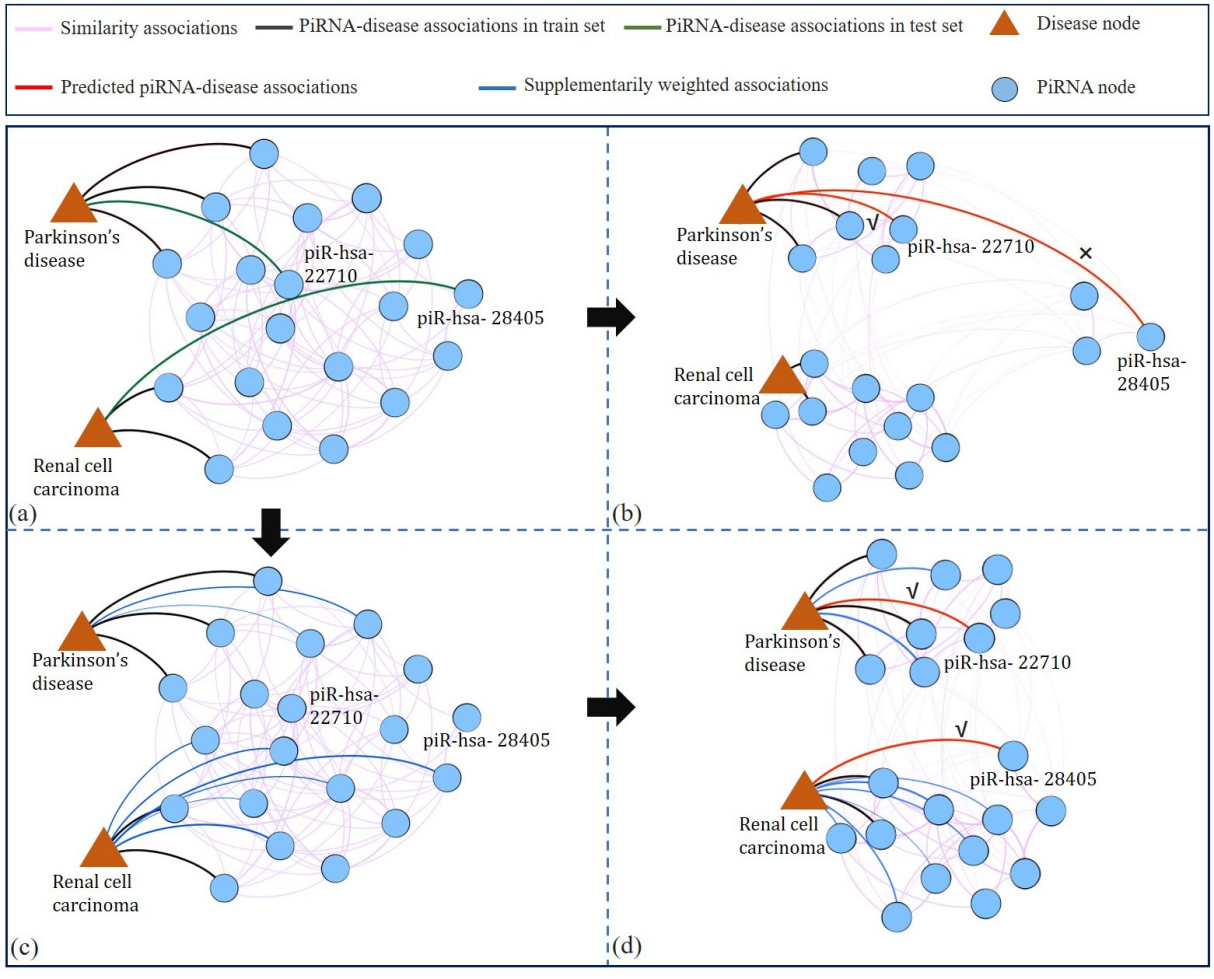
The prediction visualization of iPiDA-SWGCN. The figures are plotted by using Gephi [61]. (a) PiRNA-disease associations for training and test. The black lines denote the associations in the training set, and the green lines denote the associations in test set for prediction. (b) Predicted results of the GCN performed on the original piRNA-disease association network. The red lines show the prediction results on the original network, and Parkinson’s disease is incorrectly predicted to be associated with piR-has-28405. (c) Supplementarily weighted piRNA-disease association network. The blue lines denote the supplementarily weighted associations computed by several basic predictors. (d) Predicted results of the GCN performed on the supplementarily weighted piRNA-disease association network. The associations in the test set are correctly predicted by GCN when adding supplementarily weighted associations.

The results are shown in Fig 5, from which we can draw the following conclusions: (i) Due to the shortage of verified piRNA-disease associations, the ability of GCN to capture network structural information is limited; (ii) GCN performing on the supplementarily weighted network can correctly predict the test piRNA-disease associations, because the weighted network cannot only provide more proximity structural information, but also contains the association confidence.

### Case study

To illuminate the practicability of iPiDA-SWGCN, we applied iPiDA-SWGCN to detect potential piRNAs associated with three important diseases (‘Renal cell carcinoma’, ‘Parkinson’s disease’ and ‘Cardiovascular disease’). The top five detected piRNAs and their corresponding literature evidence are listed in Table 4. From Table 4 we can see that all the top five detected piRNAs associated with ‘Renal cell carcinoma’ and ‘Parkinson’s disease’ have been validated by literatures. Four of five detected piRNAs associated with ‘cardiovascular disease’ are confirmed by the literature. For example, piR-hsa-23184 shows a higher expression of 2.26-fold in metastatic compared to non-metastatic tumor [60]. The piR-hsa-5389 is upregulated in cells and post-mortem tissue samples between control and Parkinson’s disease patients [59]. The expression of piR-hsa-25177 in cardio sphere cells is 3.38-fold higher than that in cardio sphere-derived cells [62]. Therefore, iPiDA-SWGCN can effectively detect potential piRNA-disease associations, which is suitable for real world applications. The more specific case study results are shown in Table B in S1 Text.

**Table 4:** The top 5 piRNAs associated with different diseases detected by iPiDA-SWGCN.

Disease	Rank	piRNA	Evidence^a^
Renal cell carcinoma	1	piR-hsa-753	PMID:26071182
	2	piR-hsa-22558	PMID:26071182
	3	piR-hsa-28427	PMID:26071182
	4	piR-hsa-23184	PMID:26071182
	5	piR-hsa-7714	PMID:26071182
Parkinson’s disease	1	piR-hsa-5389	PMID:29986767
	2	piR-hsa-26242	PMID:29986767
	3	piR-hsa-24656	PMID:29986767
	4	piR-hsa-1271	PMID:29986767
	5	piR-hsa-14134	PMID:29986767
Cardiovascular disease	1	piR-hsa-25177	PMID:27131603
	2	piR-hsa-30122	PMID:27131603
	3	piR-hsa-3578	PMID:27131603
	4	piR-hsa-1180	PMID:27131603
	5	piR-hsa-19768	PMID:27131603

^a^The identified piRNA-disease associations are confirmed by the literature. The PMIDs for the evidence literature are given as well.

## Conclusion

In this work, we propose a novel predictor named iPiDA-SWGCN for piRNA-disease association prediction by combining the supplementarily weighted strategy and GCN. The iPiDA-SWGCN mainly has following advantages: (i) Potential piRNA-disease associations are supplemented in the piRNA-disease network by integrating various basic predictors to provide an informative network, based on which GCN can capture deep proximity structure, and fully utilize network information for feature learning. (ii) Different confidences are assigned to the piRNA-disease associations instead of Boolean values. Therefore, GCN aggregates node information and accurately learns node representations from neighbor nodes in varying degrees. (iii) Although iPiDA-SWGCN is proposed for predicting piRNA-disease associations, it can be extended to other link prediction tasks.

It is anticipated that the strategy of supplementarily weighted adjacency matrix will be applied to other related fields to solve the problems of limited positive samples, such as lncRNA–disease association detection and drug repositioning. In particular, there are plenty of unknown associations in link prediction field resulting in high sparsity problem. The supplementarily weighted strategy can be implemented to preliminarily enrich the association network so as to provide more useful information and improve the prediction performance.

Besides, the value of piRNA-disease associations without independent experimental validation is worth mentioning. PiRNAs are potential biomarkers that may provide new avenues of investigation into the pathogenesis of diseases. However, it should be noted that these associations predicted by iPiDA-SWGCN are only correlations and require experimental validation to confirm their significance. This is especially relevant for piRNA studies due to their high abundance and poorly understood roles in diseases.

Future studies should aim to validate these associations through a combination of biological experiments and bioinformatics analysis to ensure their reliability and accuracy, promoting to gain a better understanding of the complex mechanisms underlying diseases and develop more effective strategies for prevention and treatment.

## Supporting information

S1 TextFig A. Parameter analysis of iPiDA-SWGCN. Table A. The performance comparison of basic predictors on $D_{ind}^{2}$. Table B. The top 10 piRNAs associated with different diseases detected by iPiDA-SWGCN.(DOCX)
