# Supplementary material for: iPiDA-SWGCN: Identification of piRNA-disease associations based on Supplementarily Weighted Graph Convolutional Network
Source: PLoS Comput Biol. 2023 Jun 20;19(6):e1011242. doi: 10.1371/journal.pcbi.1011242 (PMC10313042; doi:10.1371/journal.pcbi.1011242)
Supplement: S1 Text — Fig A. Parameter analysis of iPiDA-SWGCN. Table A. The performance comparison of basic predictors on Dind2. Table B. The top 10 piRNAs associated with different diseases detected by iPiDA-SWGCN. (DOCX) [file pcbi.1011242.s001.docx]

Supplementary material

We conduct experiments to analyze the impact of four parameters in our proposed model based on the benchmark dataset. The benchmark dataset is divided into five folds, and the model was trained on four folds while one-fold was used for testing. The parameter analysis experiments are performed by varying one parameter while the other parameters fixed. The results are shown in **Fig A**.

**GCN layer**

As shown in **Fig A(a)**, with the number of GCN layer increasing, GCN can aggregate more neighborhood information and achieve the optimal performance with two GCN layers. However, further deepening the GCN layers leads to over-smoothing and indiscriminative node features, causing performance decrement. Therefore, the number of GCN layers is set to 2.

**Learning rate**

The learning rate is a critical parameter that can adjust the step size to update model weights in each iteration during training process. A small learning rate make the modelconverge slowly, while a large learning rate may cause divergence or oscillated around the optimal solution. **Fig A(b)** shows that there is an optimal value for the initial learning rate, thuswe set the learning rate to 0.001.

**Epoch**

As shown in **Fig A(c)**, the performance of our model initially improves and then decreases with the increment of epochs attributed to overfitting. As the number of epochs keep increasing, the model continues to fit the training data more and more closely, resulting in decrement of generalization ability. In this study, the number of epochs is set as 1000.

**Weight decay factor**

The weight decay factor is a regularization technique in deep learning, which adds a regularization term to the cost function to prevent overfitting. The performace influenced by weight decay factor is shown in **Fig A(d)**. In this study, the weight decay factor is set as 1.


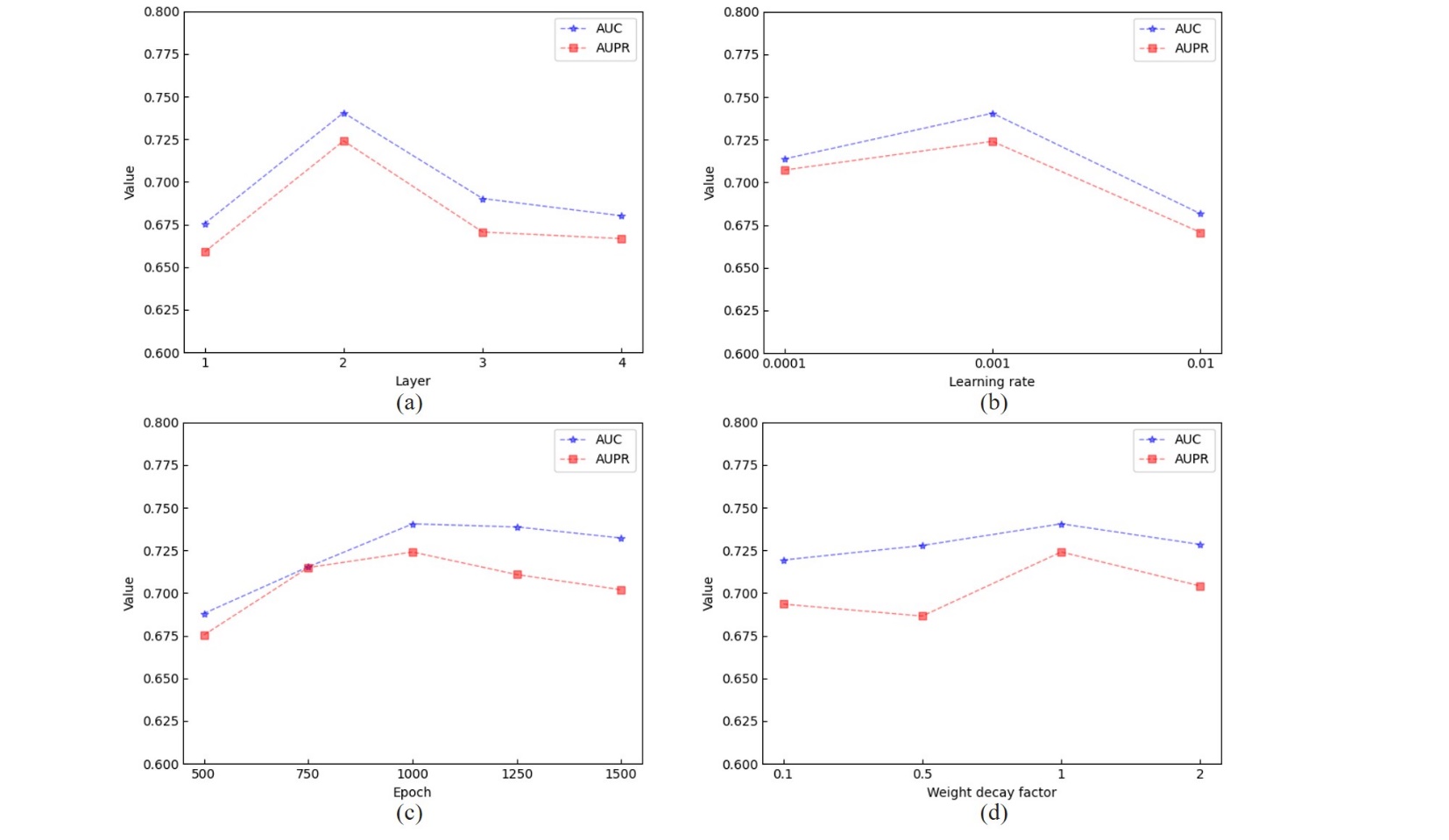
**Fig A.** Parameter analysis of iPiDA-SWGCN. The influence of GCN layer, learning rate, epoch and weight decay factor on the performance of iPiDA-SWGCN is shown in (a), (b), (c) and (d), respectively.

**Table A** The performance comparison of basic predictors on $D_{ind}^{2}$

| **Method** | **AUC** | **AUPR** |
| --- | --- | --- |
| SVM | 0.6605 | 0.6468 |
| RF | 0.7007 | 0.6732 |
| GBDT | 0.6939 | 0.6704 |
| Basic predictor combination | 0.7078 | 0.6825 |
| **iPiDA-SWGCN** | **0.8178** | **0.8151** |

**Table A** shows the basic predictors performance in terms of AUC and AUPR. From which we can see that the SVM and RF achieve the lowest and highest performance of individual predictor respectively, and the combination of basic predictors help to improve the basic predictor performance.

**Table B** The top 10 piRNAs associated with different diseases detected by iPiDA-SWGCN.

| **Disease** | **Rank** | **piRNA** | **Evidence^a^** | **Regulation^b^** |
| --- | --- | --- | --- | --- |
| Renal cell carcinoma | 1 | piR-hsa-753 | PMID:26071182 | up |
|  | 2 | piR-hsa-22558 | PMID:26071182 | up |
|  | 3 | piR-hsa-28427 | PMID:26071182 | down |
|  | 4 | piR-hsa-23184 | PMID:26071182 | up |
|  | 5 | piR-hsa-7714 | PMID:26071182 | up |
|  | 6 | piR-hsa-13637 | PMID:26071182 | down |
|  | 7 | piR-hsa-18404 | PMID:26071182 | up |
|  | 8 | piR-hsa-5447 | PMID:26071182 | up |
|  | 9 | piR-hsa-20006 | PMID:26071182 | down |
|  | 10 | piR-hsa-13462 | PMID:26071182 | down |
| Parkinson’s disease | 1 | piR-hsa-5389 | PMID:29986767 | up |
|  | 2 | piR-hsa-26242 | PMID:29986767 | down |
|  | 3 | piR-hsa-24656 | PMID:29986767 | up |
|  | 4 | piR-hsa-1271 | PMID:29986767 | down |
|  | 5 | piR-hsa-14134 | PMID:29986767 | down |
|  | 6 | piR-hsa-6328 | PMID:29986767 | down |
|  | 7 | piR-hsa-30054 | PMID:29986767 | down |
|  | 8 | piR-hsa-7837 | PMID:29986767 | down |
|  | 9 | piR-hsa-2122 | PMID:29986767 | down |
|  | 10 | piR-hsa-4978 | PMID:29986767 | up |
| Cardiovascular disease | 1 | piR-hsa-25177 | PMID:27131603 | up |
|  | 2 | piR-hsa-30122 | PMID:27131603 | up |
|  | 3 | piR-hsa-3578 | PMID:27131603 | up |
|  | 4 | piR-hsa-1180 | PMID:27131603 | up |
|  | 5 | piR-hsa-19768 | PMID:27131603 | up |
|  | 6 | piR-hsa-18505 | PMID:27131603 | up |
|  | 7 | piR-hsa-22335 | PMID:27131603 | up |
|  | 8 | piR-hsa-18504 | PMID:27131603 | up |
|  | 9 | piR-hsa-20678 | PMID:27131603 | up |
|  | 10 | piR-hsa-18501 | PMID:27131603 | up |
| Alzheimer’s disease | 1 | piR-hsa-28488 | PMID:28127595 | up |
|  | 2 | piR-hsa-23289 | PMID:28127595 | up |
|  | 3 | piR-hsa-4946 | Unconfirmed | NA |
|  | 4 | piR-hsa-7238 | Unconfirmed | NA |
|  | 5 | piR-hsa-28489 | PMID:28127595 | up |
|  | 6 | piR-hsa-1580 | PMID:28654860 | up |
|  | 7 | piR-hsa-24680 | PMID:28127595 | up |
|  | 8 | piR-hsa-1344 | Unconfirmed | NA |
|  | 9 | piR-hsa-32299 | Unconfirmed | NA |
|  | 10 | piR-hsa-23231 | PMID:28127595 | up |

^a^ The identified piRNA-disease associations confirmed by the literature. The PMIDs for the evidence literature are given as well.

^b^ The piRNA is differently up-expressed or down-expressed in the target disease.

Case studies on four diseases are implemented, including Renal cell carcinoma, Parkinson’s disease, cardiovascular disease and Alzheimer’s disease. Renal cell carcinoma (RCC) is a prevalent cancer, ranking sixth in incidence in men and tenth in women globally [1]. Multiple studies have demonstrated that piRNAs show varying expression levels in benign and malignant renal tissues [2, 3]. Parkinson's Disease (PD) is a widely prevalent neurodegenerative disorder, ranking second in frequency globally. Recent study indicates that some piRNA gene targets were enriched for neurodegenerative disease and specific genes identified highlighted pseudogenes related to Parkinson’s disease [4]. The morbidity and mortality of cardiovascular disease (CVD) have risen continuously in recent years, with a gradually decreasing age of onset and a mortality rate higher than any other disease [5]. Alzheimer's disease (AD) is a progressive neurodegenerative disorder and the main cause of dementia among the elderly worldwide [6]. With the development of research in piRNAs and Alzheimer's disease, it is proved that piRNAs are abundant in human brains and may represent risk biomarkers of AD [7].

Predicted top 10 candidate piRNAs related to each disease are shown in **Table B**, from which we can see that most predicted associations can be validated by literature. For example, piR-hsa-13637 shows a lower expression of 20.81-fold in metastatic compared to non-metastatic tumor while piR-hsa-18404 shows a 49.81-fold up-regulated expression [8]. The piR-hsa-6328 has a 1.68-fold upregulated expression in cells and post-mortem tissue samples between control and Parkinson’s disease patients [9]. The expression of piR-hsa-22335 in cardio sphere cells is 2.66-fold higher than that in cardio sphere-derived cells [10]. The expression of piR-hsa-28488 is 3.94-fold higher in Alzheimer's disease-affected brain compared with the normal human brain [11].

Reference

1. Capitanio U, Bensalah K, Bex A, Boorjian SA, Bray F, Coleman J, et al. Epidemiology of renal cell carcinoma. European urology. 2019;75(1):74-84.

2. Li Y, Wu X, Gao H, Jin JM, Li AX, Kim YS, et al. Piwi-interacting RNAs (piRNAs) are dysregulated in renal cell carcinoma and associated with tumor metastasis and cancer-specific survival. Molecular Medicine. 2015;21:381-8.

3. Zhao C, Tolkach Y, Schmidt D, Toma M, Muders MH, Kristiansen G, et al. Mitochondrial PIWI-interacting RNAs are novel biomarkers for clear cell renal cell carcinoma. World Journal of Urology. 2019;37:1639-47.

4. Zhang T, Wong G. Dysregulation of human somatic piRNA expression in Parkinson’s disease subtypes and stages. International Journal of Molecular Sciences. 2022;23(5):2469.

5. Zeng Q, Cai J, Wan H, Zhao S, Tan Y, Zhang C, et al. PIWI-interacting RNAs and PIWI proteins in diabetes and cardiovascular disease: Molecular pathogenesis and role as biomarkers. Clinica Chimica Acta. 2021;518:33-7.

6. Jain G, Stuendl A, Rao P, Berulava T, Pena Centeno T, Kaurani L, et al. A combined miRNA–piRNA signature to detect Alzheimer’s disease. Translational psychiatry. 2019;9(1):250.

7. Qiu W, Guo X, Lin X, Yang Q, Zhang W, Zhang Y, et al. Transcriptome-wide piRNA profiling in human brains of Alzheimer's disease. Neurobiology of aging. 2017;57:170-7.

8. Busch J, Ralla B, Jung M, Wotschofsky Z, Trujillo-Arribas E, Schwabe P, et al. Piwi-interacting RNAs as novel prognostic markers in clear cell renal cell carcinomas. Journal of experimental & clinical cancer research. 2015;34(1):1-11.

9. Schulze M, Sommer A, Plötz S, Farrell M, Winner B, Grosch J, et al. Sporadic Parkinson’s disease derived neuronal cells show disease-specific mRNA and small RNA signatures with abundant deregulation of piRNAs. Acta neuropathologica communications. 2018;6(1):1-18.

10. Vella S, Gallo A, Nigro AL, Galvagno D, Raffa GM, Pilato M, et al. PIWI-interacting RNA (piRNA) signatures in human cardiac progenitor cells. The international journal of biochemistry & cell biology. 2016;76:1-11.

11. Roy J, Sarkar A, Parida S, Ghosh Z, Mallick B. Small RNA sequencing revealed dysregulated piRNAs in Alzheimer's disease and their probable role in pathogenesis. Molecular BioSystems. 2017;13(3):565-76.
